# Description of familial keloids in five pedigrees: evidence for autosomal dominant inheritance and phenotypic heterogeneity

**DOI:** 10.1186/1471-5945-9-8

**Published:** 2009-07-28

**Authors:** Jason A Clark, Maria L Turner, Lillian Howard, Horia Stanescu, Robert Kleta, Jeffrey B Kopp

**Affiliations:** 1 Kidney Disease Section, National Institute of Diabetes and Digestive and Kidney Diseases, National Institutes of Health, Bethesda, MD, USA; 2 Dermatology Branch, National Cancer Institute, National Institutes of Health, Bethesda, MD, USA; 3 Department of Medicine, University College London, UK

## Abstract

**Background:**

Familial keloids have been reported, having either autosomal dominant or autosomal recessive inheritance. We wished to determine the inheritance pattern and phenotype of keloids among multigenerational families, as a prelude to a positional mapping strategy to identify candidate genes.

**Methods:**

We studied three African American families, one Afro-Caribbean family and one Asian-American family. Phenotyping including assessing all patients for the presence, distribution, and appearance of keloids, together with the timing of keloid onset and provocative factors. The clinical trial was registered at clinicaltrials.gov (NCT 00005802).

**Results:**

Age of keloid onset varied considerably within families, but commonly occurred by the second decade. The fraction of affected individuals was 38%, 45%, 62%, 67% and 73% among the five families respectively. Keloid severity and morphology differed within and between families. A novel finding is that certain families manifest keloids in distinct locations, with one family showing an excess of extremity keloids and two families showing an excess of axilla-groin keloids.

**Conclusion:**

Familial keloids appear to most commonly manifest autosomal dominant or semidominant inheritance, and there may be familial patterns of keloid distribution.

## Background

Keloids represent an exuberant wound healing response that may occur spontaneously or following cutaneous injury. Worldwide keloid prevalence varies by geographic ancestry from 0.09% in Great Britain to 16% in the Congo [1]. Although keloid prevalence in United States is not well documented, darker-skinned individuals and those of African descent seem to be disproportionately affected [2]. Keloid scars can impair physical and psychological quality of life [3]. Available therapies have low efficacy and/or significant morbidity. The International Clinical Recommendations on Scar Management list a variety of therapeutic approaches including triamcinolone, surgery, radiation, and combination therapy [4]. A recent prospective study found greater than 70% recurrence at a mean follow up of 19 months for surgical excision followed by irradiation [5]. Surgical resection with concomitant triamcinolone injection, is followed by a recurrence rate of up to 20% and the recurrent lesion is often worse than the original lesion [6]. Other therapies including intralesional 5-fluorouracil, calcium channel blockers, bleomycin, cyclosporine, topical retinoic acid and imiquimod have shown some promise in the treatment of keloids, yet none consistently outperform surgery with triamcinolone injection [6].

Keloids and hypertrophic scars are distinct clinically and pathologically. Keloids may appear spontaneously or following trauma, expand beyond the margins of the wound, and typically persist and expand for a number of years. Hypertrophic scars occur following trauma, are limited to the boundaries of the wound, and typically begin to regress within one year of injury. Histologically, keloids are characterized by a greatly expanded dermis occupied by large, hyalinized collagen fibers that are strongly eosinophilic, while hypertrophic scars are characterized by an expanded dermis, with more numerous fibroblasts and thin, newly deposited collagen fibers. Both keloids and hypertrophic scars show dermal nodules comprised of focal aggregates of fibroblasts together with randomly oriented collagen fibers. In hypertrophic scars, the dermal nodules have well-demarcated borders while in keloids the borders are less distinct. In contrast to both keloids and hypertrophic scars, normal scars lack dermal nodules, contain loose aggregates of fibroblasts arranged in parallel to the epidermis, and exhibit less conspicuous collagen fibers. Ehrlich *et al*. demonstrated that alpha-smooth muscle actin expressing myofibroblasts are specific to hypertrophic scars and are absent from normal scars and keloids [7]. Burd and Huang, however, highlight discrepancies among histologic descriptions of keloids and hypertrophic scars, concluding that histology changes through time and may also vary within a scar at a single time point [8].

Numerous theories have been proposed for the pathogenesis of keloids. [6] Recently the role of TGF-beta, a cytokine important in wound healing and fibrosis, has been explored in keloids. Cultured keloid fibroblasts demonstrate increased fibroblast proliferation, TGF-beta expression, and collagen production. Xia and colleagues showed that TGF-beta2 production increases following serum stimulation of keloid fibroblasts [9]. Moreover, Sato demonstrated that keloid fibroblasts express more beta-catenin, a TGF-beta downstream effector molecule [10]. Further, expression of SMAD6 and SMAD7, which terminate TGF-beta signaling, is downregulated in keloid tissue. On the other hand, Bayat and colleagues found that plasma TGF-beta1 levels did not vary significantly between Caucasian patients with keloids, hypertrophic scars and controls and that polymorphisms in *TGFB1, TGFB2*, and *TGFBR1 *were not associated with keloids or hypertrophic scars [11-13].

Three investigative groups, working with diverse racial and ethnic populations, have concluded that familial keloids manifest autosomal dominant inheritance. Bloom reported 31 mostly European keloid pedigrees, including one of African origin[14]; Marneros *et al*. reported 14 American keloid pedigrees [15]; and Chen *et al*. reported 6 Han Chinese pedigrees [16]. Omo-Dare, *et al*. however, analyzed 34 pedigrees in Nigeria and concluded that the data indicated recessive inheritance [17]. Recently, genetic loci have been identified in two families with autosomal dominant keloids, at 2q23 (Japanese family) and 7p11 (African-American family) [18]. Both loci were subsequently excluded in two Han Chinese families with keloids; preliminary data from one of these families suggests potential linkage at 10q23.31 [19].

Keloids have been reported in association with two genetic syndromes. Rubinstein-Taybi syndrome manifests beaked nose, widened terminal phalanges of the thumbs and great toes, and mental retardation. In a review, Siraganian *et al*. reported keloids in 28 of 574 cases [20]. The syndrome has been linked to a variety of mutations at 16p13.3 (*CBP*), the gene that codes for the cAMP response element binding (CREB) protein. *CBP *functions in multiple signal transduction pathways and is thought to regulate the expression of many genes [21]. Goeminne syndrome is characterized by torticollis, cryptorchidism, renal dysplasia and multiple nevi. Three of the seven reported cases had keloids. The syndrome has been associated with balanced X/autosome translocation and mapped to locus Xq28 [22].

We set out to further characterize the genetics of non-syndromic familial keloids in the US population. We studied five families each with three or more affected members with at least two affected generations.

## Methods

### Subjects

The Institutional Review Board of the National Institute of Diabetes, Digestive, and Kidney Diseases, National Institutes of Health (NIH) approved the research protocol, which has been listed at  since 2001. Investigators recruited patients from the dermatology and plastic surgery departments of a nearby teaching hospital. In addition, investigators sent letters describing the study to local dermatologists and plastic surgeons. Finally with IRB approval, NIH Patient Recruitment Office placed advertisements in local newspapers. Many subjects learned about the study either by calling NIH to inquire about keloid studies or by finding the research protocol on the Internet. Subjects with keloids contacted study investigators, and those with at least three affected family members were asked to participate in family studies. Subjects over 18 yrs of age gave informed consent; subjects <18 yrs of age gave informed assent and their parents or guardians gave informed consent to participate in these studies. All probands resided within the US. Using phone interviews, partial pedigrees were assembled for 13 families, and five families were selected for further study, based exclusively on the availability and willingness of family members to participate. Geographic ancestry (racial and ethnic background) was determined by self-report.

For the five families presented here, detailed pedigrees were constructed. Affected individuals who were determined by telephone interview to have keloids or possible keloids were invited to travel to the NIH Clinical Center. Nineteen of 35 affected subjects from the five families were examined by a dermatologist (MT). An additional seven affected subjects were examined in their home states and photographs were taken for review by a dermatologist. No kindreds had evidence for Rubinstein-Taybi or Goeminne syndromes on clinical exam. Ten affected subjects were not available for examination. Of these, five subjects were deceased, three subjects could not travel (reasons included pregnancy, residence abroad and work schedule), one subject refused, and one subject could not be located by family members.

The clinical diagnosis of keloid was based on the following criteria: a scar which extended beyond original wound margins together with a history of continued scar growth more than 12 months after appearance of the lesion. Biopsy was not performed, due to risk of keloid exacerbation. Hypertrophic scar was defined as a raised scar that was confined to the boundaries of the original wound and which reached maximum extent within 12 months of onset, following by a period of slow regression. Photographs were taken of all keloids as well as many hypertrophic scars and normal scars.

The clinical ascertainment of unaffected family members was generally made by self-identification, (individuals stated that they had no unusual scars). Nine unaffected subjects were examined at the NIH Clinical Center or in their home state. When a subject was unsure as to whether a scar was unusual and might represent a keloid, he or she was examined at the NIH Clinical Center or in their home state; five such patients were diagnosed with hypertrophic scars. Blood was obtained from both affected and unaffected subjects for a whole genome scan, which is underway at present.

### Analysis of pedigrees

**Figure 1:**
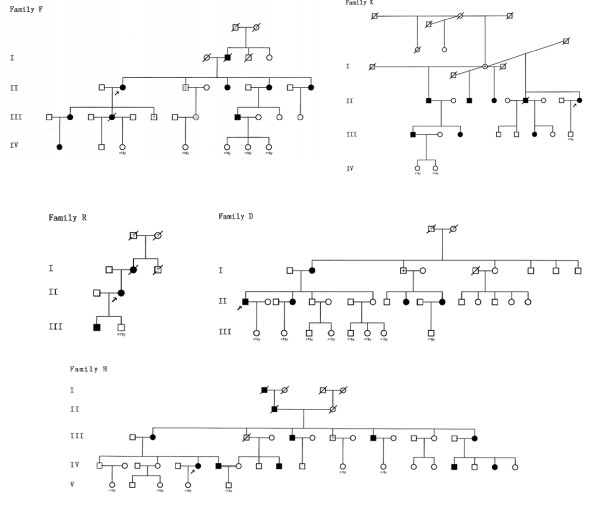
**Pedigrees of five families with familial keloids**. Pedigrees for the five keloid families (D, F, H, K, and R) are shown. Notable features include the following: vertical transmission without consanguinity, male-to-male transmission, and approximately equal numbers of affected and unaffected individuals. Taken together, these findings support dominant or semidominant inheritance. Generations available for study are numbered with Roman numerals. Black symbols indicate keloids or keloids plus hypertrophic scar; gray symbols indicate hypertrophic scar only. Arrows denote probands. Question marks indicate individuals who were not available for study; this includes two individuals in the numbered generations. A dot identifies the three obligate carriers, assuming autosomal dominant inheritance.

We prepared pedigrees for the five families (Figure 1). All family members are shown in these pedigrees including 2 subjects whose clinical status is unknown and 23 children <18 yrs old. We also prepared a tabulation of family members with keloids, hypertrophic scars, and neither skin disease (Table 1). Keloids frequently appear for the first time during adolescence and it is not possible to reliably determine keloid phenotype in children. Therefore, we excluded all individuals <18 yrs from Table 1 as the inclusion of these individuals would likely mis-categorize some children as unaffected who will manifest keloids later in life. In cases where individuals were deceased prior to study onset, keloid status was assigned based on report by family members. When possible, multiple family members were contacted to confirm phenotype. When family members did not know the phenotype of a deceased relative, these individuals were characterized as having unknown keloid status. The number of generations affected was determined based on the first generation manifesting keloids or an obligate carrier and the last generation with at least one individual over 18 years of age with or without keloids. Tabulations were calculated across affected generations.

**Table 1:** Characteristics of five families with familial keloids

Family	Ethnicity	Generations studied	Subjects	Affected subjects (keloids)	Unaffected subjects (with hypertrophic scars)	Unaffected Subjects (no abnormal scars)	Unknown status
F	African American	4	15	9	2	3	1
K	African American	3	12	8	0	4	0
R	African American	3	4	3	1	0	0
D	Afro-Caribbean	2	18	5	0	13	0
H	East Indian	5	24	11	1	11	1
Total		17	73	36	4	31	2

The characteristics of the five families with familial keloids are shown, including 70 family members overall. Two patients in the study could not be contacted to determine presence or absence of keloids. Children <18 yrs are excluded from this table as keloids often appear during adolescence.

### Analysis of keloid distribution and appearance

Information was recorded about each cutaneous lesion, with particular emphasis on keloids and hypertrophic scars; this included age of onset, inciting injury or event, location, treatment (if any), symptomatology, and clinical progression or regression over time. If an age range was reported, the mean was calculated, rounded to the nearest whole number and recorded as onset age. Lesions were photographed.

We determined the number and location of keloids, without regard to size, in all patients. To simplify tabulation and to limit the effect of a large number of keloids on the overall family count for that location, we counted a maximum of 5 keloids per location in each subject. We categorized keloid location according to 13 anatomic areas: head (other than ears), ears (all aspects of the external ear), shoulder (axillary fold to inferior deltoid head), upper arm (inferior deltoid head to antecubital fossa), lower arm (antecubital fossa to ulnar head), hand (distal to ulnar head), chest (sternal notch to xiphoid process to anterior axillary line bilaterally), upper abdomen (xiphoid process to umbilicus to mid axillary line bilaterally), groin (including the lower abdomen and genital region), back (C2 vertebra to L3 vertebra), buttock (L4 vertebra to gluteal folds), leg (gluteal folds to medial malleolus), and foot (distal to medial malleolus).

### Statistics

Summary statistics include mean and mode. Kruskal-Wallis testing was used to compare the age of onset of keloids among families; comparison of keloid distribution was by chi square test (3 × 2 table) or Fisher exact test (2 × 2 table), and comparison of keloid distribution by sex was by Fisher exact test. Statistical analyses were performed with Prism and InStat software (GraphPad, San Diego, CA). A p value < 0.05 was accepted as significant.

## Results and Discussion

Probands from four families (D, F, H, and K) contacted NIH after learning about the study from . The proband from family R was referred to investigators by an NIH employee. Of 17 generations represented in the 5 families, only two generations lacked an affected individual. This suggested that the mode of inheritance might be dominant or semidominant. Further, two of five pedigrees (H and K) showed evidence of male-to-male transmission, arguing against X-linked inheritance. Among individuals with a keloid-affected or status-unknown parent, the prevalence of keloids was as follows: family D 38%, family H 45%, family F 62%, family R 67%, and family K 73% (assuming obligate carrier generation I, Figure 1). These values, averaging 53%, also suggest dominant or semidominant inheritance rather than recessive inheritance. Taking all families together, adult males (15/36) and adult females (21/36) were equally affected by keloids (P = 0.24).

There were three instances in which unaffected individuals produced offspring with keloids (D, H, and K). If the inheritance pattern is dominant or semidominant, the male in generation III of family H with an affected father, siblings, and son likely represents an individual with an allele for keloids without manifesting lesions, an obligate carrier. Similarly the male in generation I of family D with an affected sister and two affected daughters and the female in generation I of family K are also likely obligate carriers. Assuming autosomal dominant inheritance, the nonpenetrance rate is 7.9%. An alternative explanation requires that offspring of these individuals underwent de novo mutation producing keloids. One, two or five separate de novo mutations would be required to explain affected offspring in family H, D and K respectively.

### Keloid characteristics

**Figure 2:**
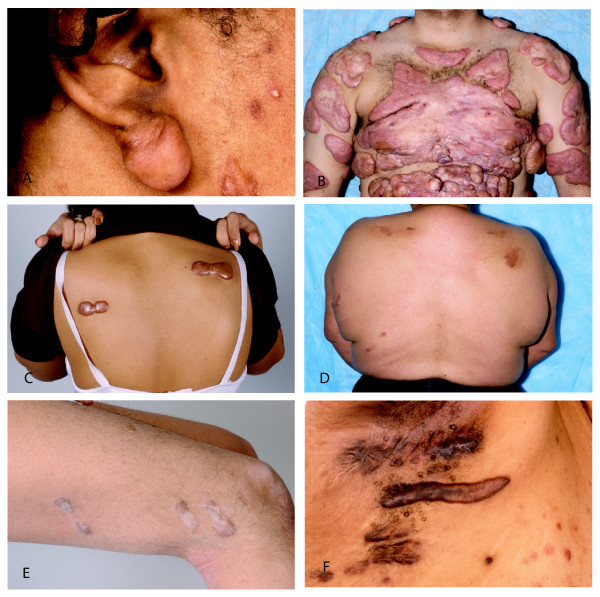
**Keloid morphology**. Keloid morphology varied within and across families. Shown is a single keloid on an earlobe and additional keloids on the cheek (A, family F), multiple keloids with areas of confluence in a severely affected individual (B, family D), classic "dumbbell" pattern keloid in the back (C, family H) and keloids on the upper back that have undergone spontaneous regression (D, family D). Certain families were more likely to show keloids in atypical locations such as extremities (E, keloids on the lateral thigh of an individual from family H) and axilla/groin (F, keloids in the axilla of an individual with hidradenitis from family F).

Keloid severity and morphology differed markedly within and among families. Lesions ranged from small earlobe nodules to multiple coalescent fibrous tumors with little intervening unaffected skin in a particular anatomic region (Figure 2A and 2B). Some lesions demonstrated the classic "butterfly" and "dumbbell" morphology, while others showed hyperpigmented geographic patches consistent with post-inflammatory hyperpigmentation at sites of reported regressed keloids (Figure 2C and 2D).

Four individuals in three families (F, H, and R Figure 1) only had hypertrophic scars; none of these individuals had keloids. All four individuals had parents with keloids or hypertrophic scars; none had children with keloids. In addition, seven subjects in 4 families (F, H, K, and R) had both keloids and hypertrophic scars.

**Figure 3:**
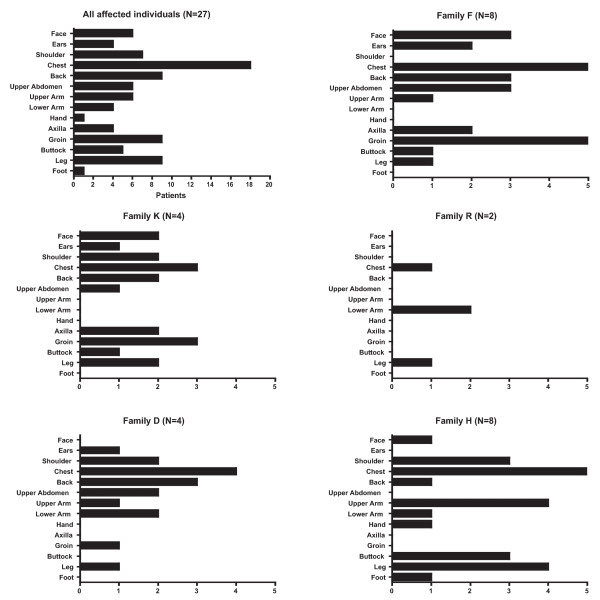
**Anatomic location of keloids**. The anatomic location of all keloids among the affected individuals of each of the 5 families and among the total group of 36 affected individuals is shown.

Keloid location also differed within and among families (Figure 3). The most frequent location for keloids was the chest (21%). Typical-location keloids, defined as those on the face, ear, chest, shoulder, back, and upper abdomen were similarly distributed among all families (p = NS). Axilla-groin keloids were more common among families F and K (10/12 individuals) compared to the other families (1/14 individuals), p < 0.001 by Fisher exact test (Figure 3). Of 10 individuals with axilla-groin keloids, two individuals from family F with multiple axillary keloids had hidradenitis on physical exam (Figure 2F). One of these subjects also had multiple groin keloids. No other subjects examined in any of the five families had hidradenitis. Four of the remaining six individuals in family F had groin keloids in association with the following sources of trauma: vasectomy, vulvar shaving, cesarean section and other surgery. All examined family members in family K had axilla-groin keloids. Two half-sisters reported multiple groin keloids following hysterectomy. Their half-brother reported a single axillary keloid following superinfected pseudofolliculitis. Another half-brother manifested a single groin keloid following surgery in addition to an axillary keloid of unknown cause. Keloid morphology varied from single nodules to multiple linear plaques (Figure 2). The increased prevalence of axillary-groin keloids was not explained by these individuals having keloids distributed all over their bodies, as most individuals had only a few keloids. Extremity keloids, defined as those on the arm, hand, buttock, leg, and foot, were more common in family H and K (10/12 individuals), rare in family F (1/7 individuals) and intermediate in families R and D (3/6 individuals), chi square test for trend p < 0.002.

**Figure 4:**
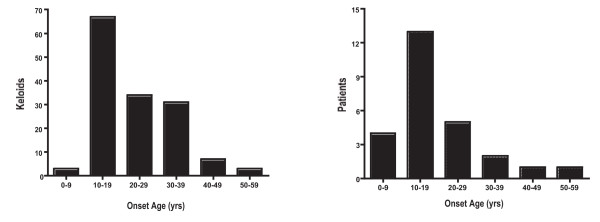
**Age of keloid onset**. A) Ages of onset of all keloids in each affected individual are shown. B) Ages of onset of first keloid in each affected individual are shown. With both analytic approaches, the modal age of onset was 10–19 years.

The age of keloid onset was obtained by history. The age reported for first keloid varied from 5 to 52 years, although most subjects examined (50%) reported onset of their first keloid between 10 and 19 years (Figure 4A). Similarly when individuals with multiple keloids were asked to recall onset age of each lesion, participants reported the largest number of keloids (46%) appearing between 10 and 19 years (Figure 4B). There were no differences among families in age of keloid appearance (p = 0.55). Of 23 subjects under 18 years of age at study end date, 14 were between 0 and 9 years of age; 9 were between 10 and 17 years. Of the nine in the latter group, four had a parent with keloids. Of the 23 subjects <18 years of age none described keloids and five were examined. None had keloids on physical exam; one, age 17, had hypertrophic scars (Figure 1, Pedigree R).

## Discussion

In the present report, we describe five pedigrees with familial keloids. Of 17 generations with adult subjects, 15 generations had at least 1 affected individual. This is consistent with autosomal dominant or semidominant inheritance given a vertical pattern of transmission, the presence of male-to-male transmission, and a ratio of approximately one half affected to non-affected persons in the absence of consanguinity. All families showed a similar propensity for keloids to affect the face, ears, and trunk, which are typical locations for both familial and sporadic keloids [16,23]. A novel finding is that certain families also manifested keloids in other locations, with one family showing an excess of extremity keloids and two families showing an excess of axilla-groin keloids.

Our findings are generally consistent with other reports indicating autosomal dominant inheritance for familial keloids. Bloom *et al*. and Marneros *et al*., together described 45 kindreds [14,15]. Of these, 34 kindreds included at least 2 generations and all were consistent with autosomal dominant inheritance. On the other hand, Omo-Dare *et al*. indicated that autosomal recessive inheritance was more likely; data on specific kindreds were not provided and so the possibility of autosomal dominant inheritance with incomplete penetrance cannot be excluded [17].

Nonpenetrance is common in autosomal dominant disorders. In the five families that are the subject of this report there were three instances where unaffected parents produced affected offspring. Assuming autosomal dominance, this suggests a nonpenetrance rate of 7.9%. Similarly, Marneros *et al*. reported a nonpenetrance rate of 6.8% after examining fourteen families with autosomal dominant inheritance [15]. Chen *et al*. reported a nonpenetrance rate of 10% after examining six autosomal dominant families [16].

Keloid phenotype varies markedly [24-26]. The present study also shows marked variability within and across families with respect to keloid number, size and severity. Past authors have ascribed these differences to variable expressivity [15,16]. Individuals within families could possess unique genetic modifiers that affect expressivity of the keloid phenotype.

The present report identified individuals in three families with hypertrophic scars and seven individuals in four families with both keloids and hypertrophic scars. Hypertrophic scars have not been previously reported in kindreds manifesting familial keloids [15,16,18]. Recent experiments cross-mating pig of different breeds, one breed manifesting a fibroproliferative scar phenotype, the other a normal scar phenotype, produced offspring with an intermediate healing phenotype [27]. In the present report, all individuals with hypertrophic scars had a parent with keloids or hypertrophic scars; none had children with keloids. It is possible that hypertrophic scars represent a milder manifestation of a keloid genetic diathesis.

Keloid location varied within and across families. While our sample, analyzed in total, suggests chest and back are the most common locations for keloids, certain families seem to show a predilection for relatively atypical areas such as axilla/groin (two African American families), and extremities (one South Asian family). Interestingly, authors have reported that of keloids of the lower limb, while relatively rare, have the strongest association with reported family history of keloids from any anatomic location [25]. Genetic susceptibility may vary by anatomic site and trends within families could reflect different mutations in the same gene, locus heterogeneity, or random variation. It remains possible that multiple genes result in keloids and individual families carry different mutations.

Familial keloids, like sporadic keloids, tended to first appear during adolescence. In our study, age at onset varied widely (5–52 years) across those sampled, yet 50% of individuals reported their first lesion between 10 and 19 years of age. Similarly, the largest number of keloids (46%) seemed to emerge during this interval. Chen reported similar results in six Han Chinese families with 53% of individuals reporting their first keloid between 11 and 20 years of age [16]. Moustafa et al. reported rapid enlargement of keloids with severe pruritus and erythema in a pregnant woman beginning at gestational month four; symptoms mitigated at delivery [28]. Together the findings suggest that hormonal milieu may influence onset and severity of both familial and sporadic keloids.

Our study has important limitations. First, subjects were identified when they contacted NIH to learn about research protocols. This approach may select for families in which at least one family member is severely affected and more likely to be searching for help. Second, our study was limited to families with at least three affected individuals, as we wished to have power for a planned genome scan. This would be expected to select for families with autosomal dominant inheritance. Thus, autosomal recessive inheritance may be more common than our study and that of others may indicate. Third, although many patients remembered trauma preceding keloids, we could not definitively distinguish spontaneous keloids from those caused by trauma. It is possible that some patients with a genetic predisposition did not develop keloids, as they had not had sufficient trauma to trigger keloid development. This might explain nonpenetrant cases. Fourth, subjects who stated they lacked keloids or unusual scars of any kind were not examined.

## Conclusion

We conclude that apparent autosomal dominant inheritance is common among multigenerational families with keloids, in families of African and South Asian ancestry. There may be differences between families in the distribution of keloids, although more studies of affected families will be required to confirm these observations.

## Competing interests

The authors declare that they have no competing interests.

## Consent

Written consent was obtained from all patients who participated in the study which was the basis for the present report, including publication of patient details.

## Authors' contributions

JC served as study coordinator, assisted by LH, and drafted the manuscript. MT evaluated patients, determined whether skin lesions had the characteristics of keloids or hypertrophic scars, and provided gross descriptions of the skin lesions. HS and RK contributed to pedigree assembly and analysis. JK conceived the study concept, oversaw the stastistical analysis and prepared the final draft of the manuscript. This study, the authors, and manuscript preparation were supported the Intramural Research Programs of the NIDDK, NCI, and NHGRI NIH. There was no commercial support for this study.

## Pre-publication history

The pre-publication history for this paper can be accessed here:


